# First in-human radiation dosimetry of the gastrin-releasing peptide (GRP) receptor antagonist ^68^Ga-NODAGA-MJ9

**DOI:** 10.1186/s13550-018-0462-9

**Published:** 2018-12-12

**Authors:** Silvano Gnesin, Francesco Cicone, Periklis Mitsakis, Axel Van der Gucht, Sébastien Baechler, Raymond Miralbell, Valentina Garibotto, Thomas Zilli, John O. Prior

**Affiliations:** 1 Institute of Radiation Physics, Lausanne University Hospital, Rue du Grand-Pré 1, 1007 Lausanne, Switzerland; 2 Department of Nuclear Medicine and Molecular Imaging, Lausanne University Hospital, Lausanne, Switzerland; 3 Department of Radiation Oncology, University Hospital of Geneva and Geneva University, Geneva, Switzerland; 4 Division of Nuclear Medicine and Molecular Imaging, University Hospital of Geneva and Geneva University, Geneva, Switzerland

**Keywords:** Gastrin-releasing peptide receptor, Bombesin, Dosimetry, PET/CT, ^68^Ga-NODAGA-MJ9, OLINDA/EXM, Theranostics, Prostate cancer, Biochemical relapse, GRP antagonist

## Abstract

**Background:**

Gastrin-releasing peptide receptor antagonists have promise in theranostics of several highly incident tumours, including prostate and breast. This study presents the first human dosimetry of ^68^Ga-NODAGA-MJ9 in the first five consecutive patients with recurrent prostate cancer included in a dual-tracer positron emission tomography (PET) protocol. Five male patients with biochemical relapse of prostate adenocarcinoma underwent four whole-body time-of-flight PET/CT scans within 2 h after tracer injection. To be used as input in OLINDA/EXM 2.0, time-integrated activity coefficients were derived from manually drawn regions of interest over the following body regions: brain, thyroid, lungs, heart, liver, gallbladder, spleen, stomach, kidneys, adrenals, red marrow, pancreas, intestines, urinary bladder and whole body. Organ absorbed doses and effective dose (ED) were calculated with OLINDA/EXM 2.0 using the NURBS voxelized phantoms adjusted to the ICRP-89 organ masses and ICRP103 tissue-weighting factors. Additional absorbed dose estimations were performed with OLINDA/EXM 1.1 to be comparable with similar previous publications.

**Results:**

The body regions receiving the highest absorbed doses were the pancreas, the urinary bladder wall, the small intestine and the kidneys (260, 69.8, 38.8 and 34.8 μGy/MBq respectively). The ED considering a 30-min urinary voiding cycle was 17.6 μSv/MBq in male patients. The increment of voiding time interval produced a significant increase of absorbed doses in bladder, prostate and testes, as well as an increase of ED. ED also increased if calculated with OLINDA/EXM 1.1. These results have been discussed in view of similar publications on bombesin analogues or on other commonly used theranostic peptides.

**Conclusions:**

The pancreas is the most irradiated organ after the injection of ^68^Ga-NODAGA-MJ9, followed by the urinary bladder wall, the small intestine and the kidneys. ED is in the same range of other common ^68^Ga-labelled peptides. Differences with similarly published studies on bombesin analogues exist, and are mainly dependent on the methodology used for absorbed dose calculations.

**Trial registration:**

Clinicaltrial.Gov identifier: NCT02111954, posted on 11/042014.

**Electronic supplementary material:**

The online version of this article (10.1186/s13550-018-0462-9) contains supplementary material, which is available to authorized users.

## Background

The amphibian skin is a quasi-unlimited source of biologically active peptides, which have been the object of extensive pharmacological studies in the past decades [1]. Most of these peptides have their counterparts in vertebrate brain and gastrointestinal tract, an occurrence that has been named as the “brain-gut-skin triangle” [2]. A striking example of such triangle is the tetradecapeptide bombesin, which was isolated from the skin of the European amphibians *Bombina bombina* and *Bombina variegata* [3] several years before the identification and sequencing of its mammalian analogue, named gastrin-releasing peptide (GRP) [4]. Yet, the name GRP might not be fully appropriate, as bombesin and its human analogue exert a wide range of biological effects, including release of hormones from gastrointestinal and endocrine organs, contraction of smooth muscles, as well as central regulation of temperature and circadian rhythms [5]. In addition, GRP modulates the function of immune cells and, most importantly, it acts as an autocrine growth factor in several tumour types, including lung [6] and prostate cancer [7]. The mitotic activity of GRP in human tumours is largely mediated by the G-protein-coupled receptor BB2, also known as GRP-receptor (GRPR); interestingly, the interference with GRPR-mediated functions produces significant anti-mitotic effects [5, 8]. It follows that the application of GRPR-targeting, radiolabelled bombesin analogues to the imaging and treatment of various neoplasms has raised considerable interest over the past 20 years [9].

Most of these research efforts have regarded prostate cancer, given the high density of GRPR since the early phase of neoplastic transformation [10]. The first radioactive bombesin analogues successfully used in prostate cancer patients were technetium-labelled GRPR agonists [11, 12]. It was later realised that, despite poor internalisation, GRPR antagonists might be advantageous over GRPR agonists because of the lack of pharmacological effects and better tumour-to-background ratios, through a higher number of binding sites or a higher affinity [13]. The increasing clinical use of positron-emitting radionuclides for tumour imaging has prompted researchers to develop newer GRPR antagonists labelled with ^68^Ga, ^18^F, or ^64^Cu [14], and it has been recently suggested that GRPR might complement prostate-specific membrane antigen (PSMA) for prostate cancer imaging [15].

The aim of the present study was to assess the human dosimetry of the GRPR antagonist ^68^Ga-NODAGA-4-amino-1-carboxymethyl-piperidine-_D_-Phe-Gln-Trp-Ala-Val-Gly-His-Sta-Leu-NH_2_ (NODAGA-MJ9) [16] in the first five patients with recurrent prostate cancer included in a dual-tracer positron emission tomography (PET) protocol.

## Materials and methods

### Patients

This dosimetry sub-study was designed to enrol the first five consecutive patients recruited in an imaging study comparing ^18^F-Fluorocholine and ^68^Ga-NODAGA-MJ9 as restaging modalities for prostate cancer in biochemical relapse (clinicaltrial.gov identifier NCT02111954, start date: April 2014). Aim of the dosimetry sub-study was to provide human absorbed dose estimations for ^68^Ga-NODAGA-MJ9, whereas dosimetry of ^18^F-Fluorocholine is already reported elsewhere [17]. Eligible patients presented a histologically confirmed prostate adenocarcinoma in biochemical relapse after a primary curative treatment (defined as prostate-specific antigen, PSA > 0.5 ng/mL after radical prostatectomy or with the nadir + 2 ng/mL definition after a primary radiotherapy [18]) for which ^18^F-Fluorocholine PET/CT was requested as restaging modality. Exclusion criteria were represented by the inability to provide written informed consent, age < 18 years and ongoing androgen deprivation therapy. The Ethical Committee of Canton Vaud, Swissmedic and the Federal Office of Public Health (FOPH), authorised the study. Patients gave separate written informed consent to the clinical and the dosimetry protocols before radiopharmaceutical administration.

### Radiochemistry

The NODAGA-MJ9 peptide was produced as a GMP product by CS Bio Co (Menlo Park, CA, USA). NODAGA-MJ9 was radiolabelled with the ^68^Ga eluate of a ^68^Ge-generator IGG100 (Eckert & Ziegler, Germany) using cassettes C4-GA68-FR on an automatic synthesis unit, Modular-Lab PharmTracer (Eckert & Ziegler, Germany). ^68^Ga was eluted with 0.1 mol/L HCl. NODAGA-MJ9 (20 μg) was radiolabelled with the high activity ^68^GaCl_3_ fraction (2 mL, 200–1300 MBq) by incubation for 20 min at room temperature. After C_18_-cartridge (Sep-Pak) pre-concentration, ^68^Ga-NODAGA-MJ9 was eluted with 50% aqueous ethanol (0.8 mL) through a 0.22 μm sterile filter, and the cartridge and filter were rinsed with sterile 0.9% NaCl solution (7 mL). High-pressure liquid chromatography (HPLC) analysis was performed at 220 nm on a μ-Bondapak column (Waters C18, 3.9 × 300 mm) by gradient elution (solvent A: 0.01 M TFA, solvent B: CH_3_CN/H_2_O:7/3 + TFA, 8/2 for 2 min then increase to 1/9 in 8 min at 1.2 mL/min). Thin-layer chromatography (TLC) was performed using sodium acetate 1 M in MeOH 1:1 on an activated iTLC-SG plate, with detection using a mini-Gita TLC scanner (Elysia-Raytest). Prior to release, all quality controls requested by the Swiss Federal authorities were met for a human injectable.

### PET/CT acquisition protocol

Four whole-body PET scans (from top of the skull to mid femoral bone, 2 min/bed position) were acquired on a Discovery 690 time-of-flight (TOF) PET/CT (GE Healthcare, Waukesha, Wisconsin, USA) 15 ± 2, 45 ± 2, 70 ± 5 and 100 ± 4  min after the intravenous administration of 113 ± 21 MBq ^68^Ga-NODAGA-MJ9. Patients were asked to void between scans (30-min voiding cycle). The list-mode acquisition integrating TOF information and point-spread-function recovery was reconstructed with a proprietary three-dimension ordered subset expectation maximisation (3D-OSEM) algorithm (GE-VPFXS, 3 iterations × 16 subsets) including a FWHM = 5 mm Gaussian post-reconstruction filter [19]. All pertinent image corrections (normalisation, dead time, activity decay, random coincidence and attenuation and scatter corrections) were applied. The acquired field of view size was 70 cm reconstructed in a 256 × 256 image matrix. Reconstructed voxel size was 2.73 × 2.73 mm in the transverse plane and 3.27 mm in the axial direction. Morphologic information was obtained from CT scan: 120 kVp, 60 mA and pitch = 3; CT FOV diameter: 700 mm; reconstructed image matrix size: 512 × 512; pixel spacing: 1.37 × 1.37 mm; and slice thickness: 3.75 mm.

As previously described, quantitative accuracy for ^68^Ga PET/CT was assessed to be within 6% of the expected value [20].

### Organ segmentation

Co-registered PET and CT data were loaded using PMOD (PMOD Technologies, Zurich, Switzerland). In PMOD, the CT matrix is used as reference for spatial resampling of PET data. Volumes of interest (VOI) were manually drawn slice by slice on the axial plane of the CT part of each PET/CT study using the polygonal segmentation tool of PMOD by two operators in consensus (SG and FC) for the following body regions: brain, thyroid, lungs, heart, liver, gallbladder, spleen, stomach, kidneys, adrenals, red marrow, intestines and whole body.

Pancreas and urinary bladder were manually segmented on the emission PET data.

CT-based segmentation of the bladder would have not taken into account possible changes of volume due to bladder filling during the PET/CT acquisition time. Manual segmentation of the pancreas based on the emission data was adopted to recover the actual organ activity by compensating for signal spill-out from the organ. No specific activity threshold was adopted for segmentation based on emission data. However, we estimated that the volume identified by our segmentation method corresponded to the volume that would result if a 5% of maximum activity threshold was adopted (data not shown).

### Absorbed dose estimations

The total activity contained in each considered source organ was obtained by multiplication of the average activity concentration (expressed in Bq/mL) by the organ volume expressed in mL. Measured activity in source organs at each time point was normalised to the administered patient activity. For all source organs but the gallbladder, a mono-exponential fit extended to infinite beyond the last measured data point was used to derive time-integrated activity coefficients (TIACs) by time-integration of source organ time-activity curves. The goodness of fit for each organ was expressed by the *R*^2^ metric. In the gallbladder, radioactivity was still in the uptake phase at the last time point image. Therefore, between *t* = 0 and *t* = 100 min, the TIAC was obtained by trapezoidal integration using Matlab software **(**Release 2017a, The MathWorks, Inc., Natick, Massachusetts, USA**)**, whereas a mono-exponential analytical integration to infinite was calculated after the last measure, assuming the ^68^Ga physical decay. This approach can be considered largely conservative because it does not take into account the physiological voiding of the gallbladder content that would naturally occur within a few hours, and that would reduce the effective tracer half-life in the organ.

Bone marrow dosimetry was estimated by drawing three red marrow VOIs, in the head of humeral bone, in the heads of the femoral bone and in the lumbar vertebrae L3-L4, respectively. The total number of disintegrations in the bone marrow was calculated by multiplying the average number of disintegrations within these three VOIs by the red marrow mass of the ICRP-89 adult male reference phantom [21]. To estimate the absorbed dose to the colon, the total number of disintegrations in this organ was partitioned to its components (right colon, left colon and rectum) proportionally to their respective masses of the ICRP-89 male reference phantom [21].

Organ TIACs were used in input to the OLINDA/EXM® 2.0 (HERMES Medical Solution AB, Stockholm, Sweden) code [22]. OLINDA/EXM 2.0 provided organ-absorbed doses and effective dose (ED) per absorbed activity in μGy/MBq and μSv/MBq, respectively, using the NURBS voxel-based phantoms [23] adjusted to the ICRP-89 organ masses [21] and ICRP103 tissue weighting factors (w_T_) [24]. Differences in organ absorbed doses estimated by varying the urinary voiding cycle in the range 0.5–3.5 h were assessed using two-sided unpaired Student’s *t* test.

By using the reference organ masses of OLINDA/EXM 2.0, we adopted a methodological approach typical of radioprotection, where the dosimetry of a reference adult subject is the major concern. Nevertheless, patient-specific dosimetry has also been performed and provided as Additional file 1: Table S1.

In order to calculate the ED for the reference person, and in view of possible applications of GRPR targeting in breast cancer [11, 25–27], we simulated organ absorbed doses and ED for female patients. Accordingly, TIACs derived from our male patients were entered in OLINDA/EXM 2.0 and applied to the organ masses of the adult female phantom. The ED for the reference person was automatically computed by the OLINDA/EXM 2.0 code using both male and female equivalent organ doses according to the ICRP 103 methodology.

### Comparison with previous studies

To facilitate the comparison with previous similar publications on dosimetry of bombesin analogues [28–34], we performed an additional absorbed dose estimation using our TIACs in input to OLINDA/EXM 1.1, which implements the Cristy & Eckerman phantoms [35] and ICRP-60 tissue w_T_ [36]. Absorbed dose calculations were performed using either a 1-h or a 3.5-h urinary voiding cycle, to allow direct comparison between our results and those of other authors using ^68^Ga-labelled bombesin analogues [30, 33, 34].

## Results

### Patients

Five prostate cancer patients (mean age 65, range 56–72 years) with biochemical relapse after radical prostatectomy with or without postoperative radiotherapy were enrolled between April and May 2014. One patient had history of pancreatic adenocarcinoma treated with pancreaticoduodenectomy and adjuvant radiochemotherapy, in clinical remission at the time of inclusion. Two patients had undergone cholecystectomy due to gallstones. Table 1 summarises patients’ characteristics and the main ^68^Ga-NODAGA-MJ9 PET/CT findings. Preliminary results of the comparison between ^18^F-Fluorocholine and ^68^Ga-NODAGA-MJ9 in the relapse setting were previously reported in an abstract form [37]. Definitive results of the main clinical study are not yet available and will be reported elsewhere.

**Table 1:** Patient characteristics

Patient	Age (years)	BMI (kg/m^2^)	Gleason score	PSA (ng/ml)	PSA doubling time (months)	^68^Ga-MJ9 PET/CT findings
1	62	31.4	9	8.5	2	Lymph nodes + bone metastases
2^a^	68	22.1	7	2.3	1	Bone metastases
3^b^	67	27.5	6	3.4	18	Bone metastases
4^b^	72	24.9	9	7.7	4.4	Lymph-nodal metastases
5	56	24.3	8	7.3	12	Local relapse

^a^History of pancreatic carcinoma treated with pancreaticoduodenectomy, and adjuvant chemo- and radiotherapy

^b^History of gallstones treated with cholecystectomy

### Radiochemistry

The ^68^Ga-NODAGA-MJ9 radiochemical purity was ≥ 95% by iTLC and < 5% free Gallium. By HPLC, the radiochemical purity was ≥ 95% ^68^Ga-NODAGA-MJ9 with a retention time of 9.0 min. There was less than 0.001% ^68^Ge radionucleidic impurity. The synthesis time of ^68^Ga-NODAGA-MJ9 was 33 min overall. The specific activity at end of synthesis (EOS) was 6–40 GBq/mg, with a peptide weight of 20 μg. The volumetric activity EOS was 15–103 MBq/mL.

### Imaging

**Fig. 1:**
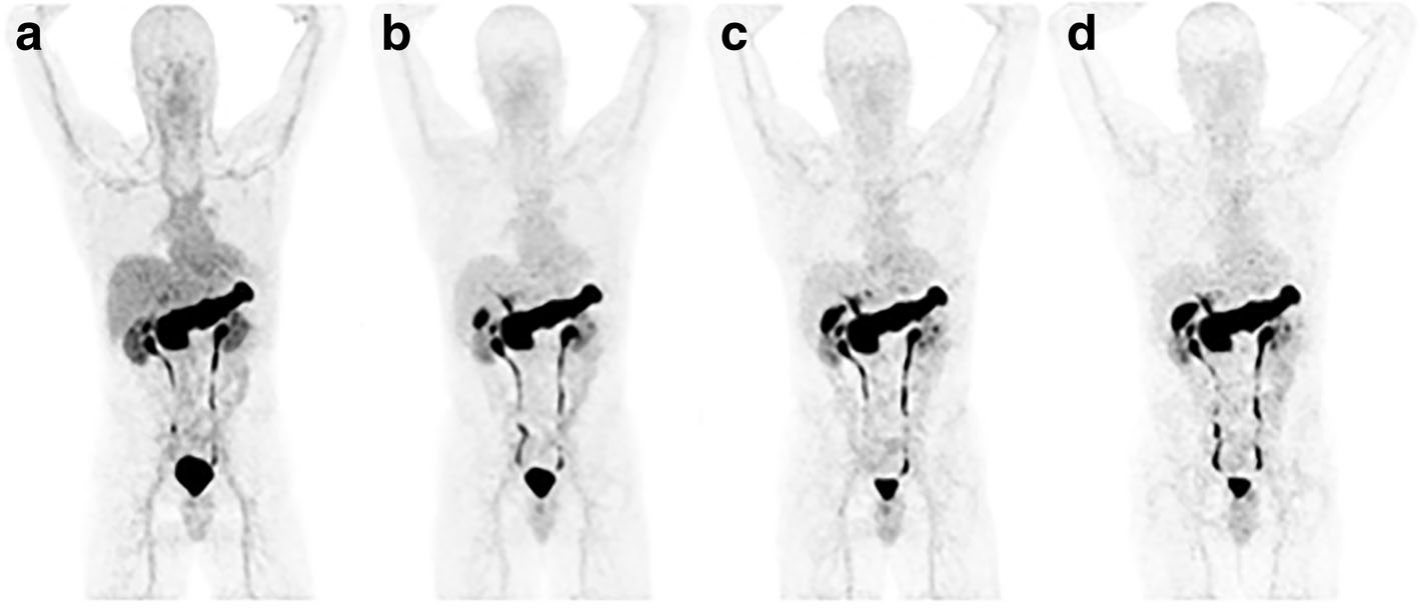
Maximum intensity projections acquired 10, 45, 70 and 100 min after ^68^Ga-NODAGA-MJ9 injection (**a**–**d**, respectively) in a 56-year-old patient with biochemical relapse of prostate cancer (patient #5). The typical ^68^Ga-NODAGA-MJ9 biodistribution is observed, including visualisation of the urinary system, as well as fast and prominent uptake by the pancreas and the biliary tract

All injections were well-tolerated. No immediate symptoms or modification of vital signs were observed. Typical biodistribution of ^68^Ga-NODAGA-MJ9 is shown in Fig. 1. The radiopharmaceutical is excreted via both urinary and hepato-biliary routes. Urinary bladder uptake is seen as early as 15 min after injection. No radiopharmaceutical retention is observed in the renal cortex, while the gallbladder is still in the biological uptake phase at the last imaging time point, 100 min after injection. A high and homogeneous pancreatic tracer uptake is seen.

### Absorbed dose estimations

**Fig. 2:**
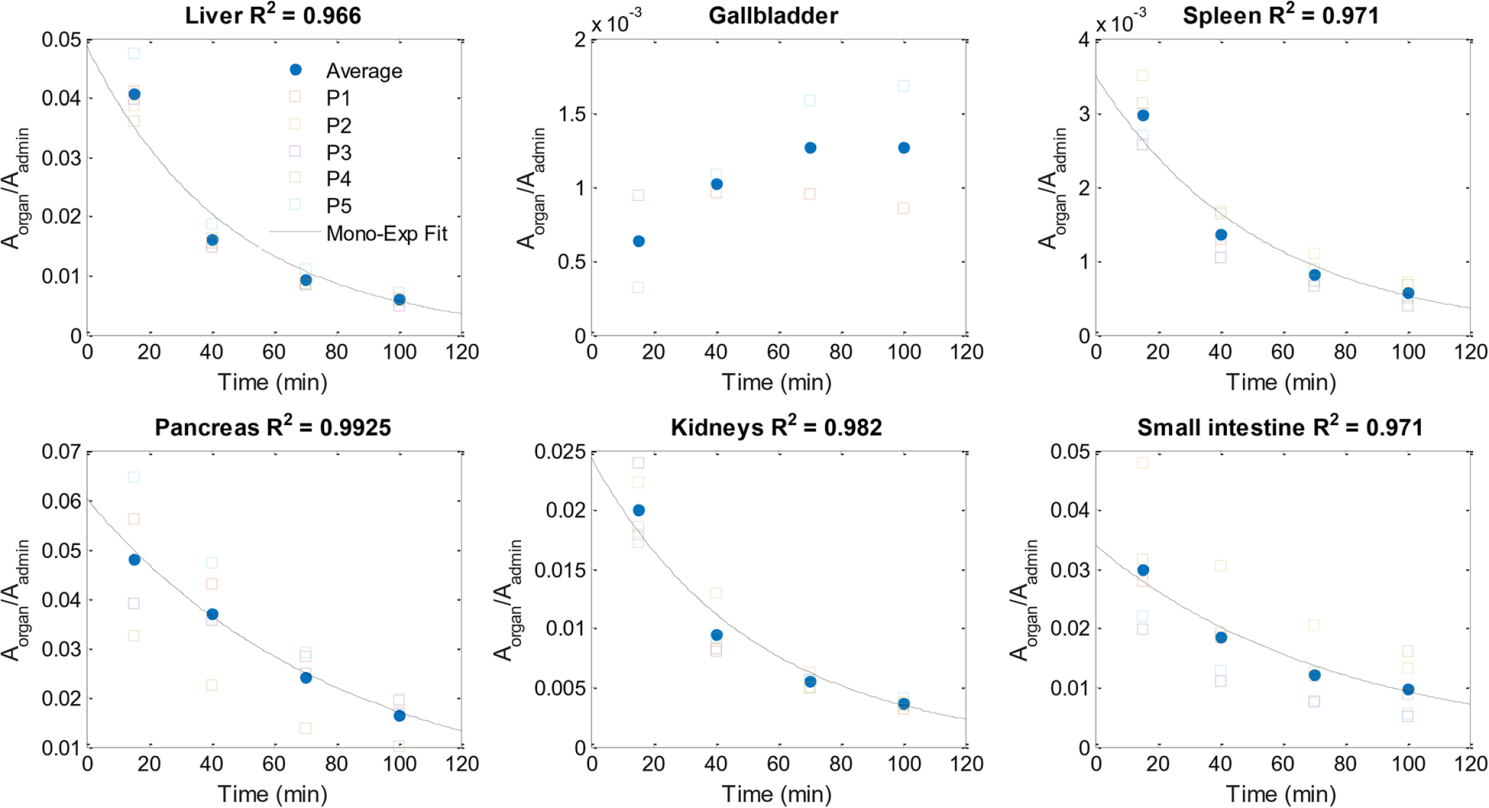
^68^Ga-NODAGA-MJ9 normalised time-activity curves for most relevant abdominal organs. The organ activity was normalised to the patient administered activity. Coloured squares indicate patient-related data points. Blue dots indicate the normalised activity (mean ± SD) obtained at each time point. The coefficient of determination (*R*^2^) of the mono-exponential fit (except for the gallbladder, see methods) is reported for each organ. Due to previous surgery, gallbladder and pancreatic normalised time-activity curves were available for two and four patients only, respectively

**Fig. 3:**
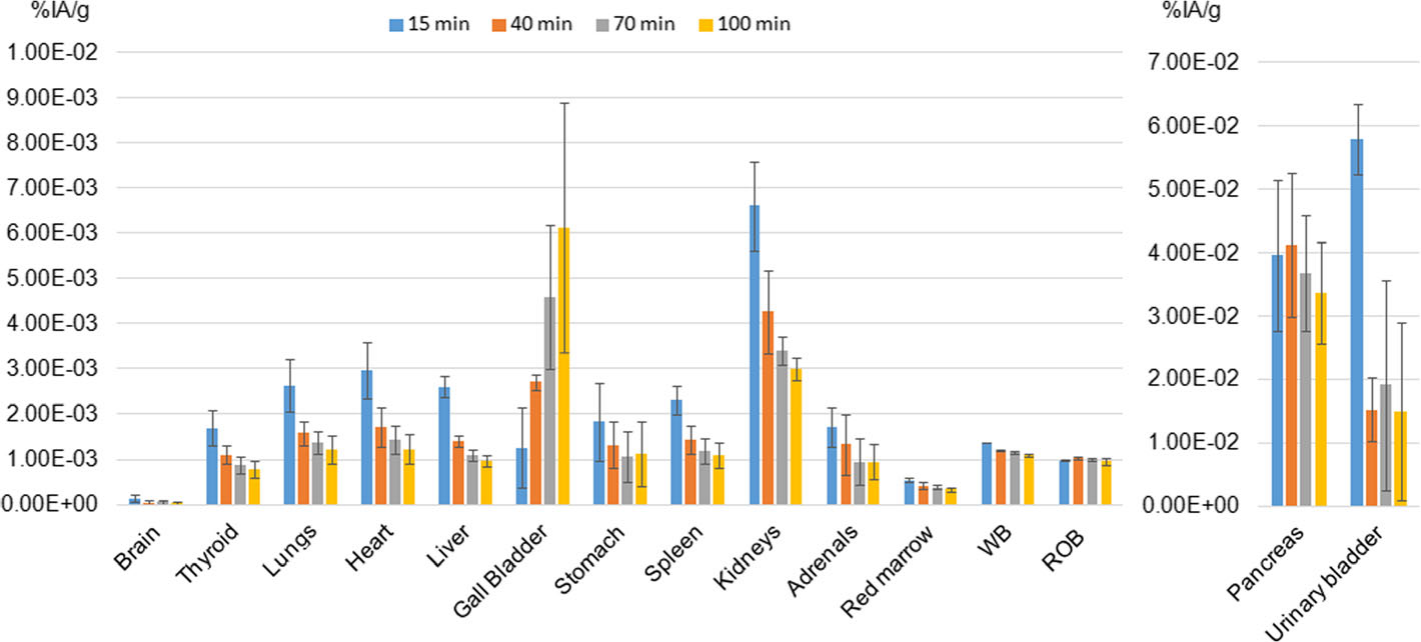
Biological organ kinetic of ^68^Ga-NODAGA-MJ9 corrected for ^68^Ga physical decay. Colour bars represent the average percent of injected activity per gram of tissue (%IA/g) ± 1SD, for each time point

Time-activity curves for relevant abdominal organs are shown in Fig. 2. Organ time-activity curves corrected for ^68^Ga physical decay are shown in Fig. 3. Measured TIACs, organ absorbed doses and ED of the patients enrolled, as well as the extrapolated values to female and reference person, are reported in Table 2.

**Table 2:** Residence times, organ-absorbed doses and ED according to OLINDA/EXM 2.0 for 30-min and 1-h urinary voiding cycles in men. Extrapolated organ-absorbed doses in female and reference person are reported for 1-h urinary voiding cycle only

Target organ	Patient cohort (*n* = 5 male)							
	Residence time (h)		Male		Male		Female	Reference
			0.5 h voiding		1 h voiding		1 h voiding	1 h voiding
	Mean	SD	Dose	SD	Dose	SD	Dose	Dose
			mGy/MBq		mGy/MBq		mGy/MBq	mGy/MBq
Adrenals	2.48E-04	8.49E-05	1.48E-02	2.22E-03	1.46E-02	2.13E-03	1.57E-02	1.52E-02
Brain	1.53E-03	1.05E-03	1.71E-03	3.12E-04	1.71E-03	3.20E-04	3.62E-03	2.68E-03
Breast	–	–	–	–	–	–	1.06E-02	1.06E-02
Esophagus	–	–	1.06E-02	5.47E-04	1.06E-02	5.22E-04	1.25E-02	1.16E-02
Eyes	–	–	8.39E-03	4.70E-04	8.39E-03	4.56E-04	1.02E-02	9.32E-03
Gallbladder wall^a^	3.60E-03	1.27E-03	2.69E-02	5.59E-03	2.69E-02	5.66E-03	2.19E-02	1.99E-02
Left colon	6.73E-03	3.33E-03	3.37E-02	9.04E-03	3.38E-02	9.01E-03	3.27E-02	3.08E-02
Small Intestine^a^	4.01E-02	1.71E-02	3.88E-02	1.05E-02	3.90E-02	1.04E-02	4.04E-02	3.76E-02
Stomach wall^a^	4.79E-03	2.45E-03	1.92E-02	1.53E-03	1.92E-02	1.53E-03	2.00E-02	1.91E-02
Right colon	1.35E-02	6.66E-03	3.21E-02	1.01E-02	3.22E-02	1.01E-02	3.28E-02	3.00E-02
Rectum	6.73E-03	3.33E-03	3.18E-02	1.02E-02	3.25E-02	9.84E-03	3.58E-02	3.17E-02
Heart wall^a^	2.16E-02	5.45E-03	2.13E-02	2.26E-03	2.13E-02	2.26E-03	2.70E-02	2.42E-02
Kidneys	2.14E-02	2.23E-03	3.48E-02	3.04E-03	3.48E-02	3.03E-03	3.31E-02	3.40E-02
Liver	3.79E-02	2.93E-03	1.41E-02	8.41E-04	1.41E-02	8.41E-04	1.85E-02	1.63E-02
Lungs	2.88E-02	5.98E-03	1.34E-02	1.95E-03	1.34E-02	1.95E-03	1.66E-02	1.50E-02
Ovaries	–	–	–	–	–	–	1.50E-02	1.49E-02
Pancreas	8.13E-02	1.95E-02	2.60E-01	6.14E-02	2.60E-01	6.14E-02	2.43E-01	–
Prostate	–	–	1.22E-02	5.52E-04	1.33E-02	7.91E-04	–	1.33E-02
Salivary glands	–	–	9.12E-03	5.06E-04	9.12E-03	4.93E-04	1.07E-02	9.93E-03
Red marrow	7.10E-03	8.59E-04	9.38E-03	4.18E-04	9.49E-03	4.06E-04	1.17E-02	1.17E-02
Osteogenic cells	–	–	6.83E-03	3.23E-04	6.89E-03	3.21E-04	7.71E-03	7.87E-03
Spleen	3.16E-03	6.73E-04	1.37E-02	1.58E-03	1.37E-02	1.58E-03	1.55E-02	1.46E-02
Testes	–	–	9.56E-03	5.28E-04	9.82E-03	5.59E-04	–	9.82E-03
Thymus	–	–	1.02E-02	3.99E-04	1.02E-02	3.60E-04	1.29E-02	1.15E-02
Thyroid	3.20E-04	5.00E-05	9.32E-03	8.30E-04	9.15E-03	8.21E-04	1.05E-02	9.85E-03
Urinary bladder wall^a^	8.77E-02	1.69E-02	6.98E-02	1.26E-02	1.12E-01	1.97E-02	1.39E-01	1.25E-01
Uterus	–	–	–	–	–	–	1.78E-02	1.76E-02
Total body	–	–	1.14E-02	5.24E-04	1.17E-02	5.87E-04	1.43E-02	1.30E-02
ED ICRP 103 (mSv/MBq)	–	–	1.76E-02	1.07E-03	1.88E-02	1.16E-03	2.30E-02	2.17E-02

^a^Irradiation from the organ content is accounted for residence time calculation

The organ receiving the highest absorbed dose was the pancreas (260 μGy/MBq), followed by the urinary bladder wall, the small intestine and the kidneys (69.8, 38.8 and 34.8 μGy/MBq respectively).

Using a 30-min urinary voiding cycle, we obtained an ED of 17.6 μSv/MBq in our male patients. The extrapolation to 1-h voiding cycle resulted in EDs of 18.8 and 23.0 μSv/MBq for male and female, respectively. The corresponding ED for the reference person was 21.7 μSv/MBq.

The increment of voiding time interval produced a significant increase of absorbed doses in the following organs: urinary bladder wall (+ 60% for 1-h vs. 0.5-h voiding cycle, *p* = 0.001, and + 186% for 3.5-h vs. 0.5-h voiding cycle, *p* < 0.0001), prostate (+ 9% for 1-h vs. 0.5-hvoiding cycle, *p* = 0.034, and + 29% for 3.5-h vs. 0.5-h voiding cycle, *p* < 0.0001) and testes (+ 2% for 1-h vs. 0.5-h voiding cycle, *p* = 0.48, and + 8.5% for 3.5-h vs. 0.5-h voiding cycle, *p* = 0.039). ED increased as well with the voiding time interval (+ 7% for 1-h vs. 0.5-h voiding cycle, *p* = 0.032, and + 33% for 3.5-h vs. 0.5-h voiding cycle, *p* < 0.0001).

### Comparison with previous studies

Results of our absorbed dose estimations performed with OLINDA/EXM 1.1 assuming 1-h and 3.5-h voiding cycles are reported as Additional file 2: Table S2.

Previously published papers on dosimetry of bombesin analogues were hardly comparable as they used various radionuclides, including ^99m^Tc [28, 29], ^64^Cu [31], ^18^F [32] or ^68^Ga [30, 33, 34], different tracer molecules, different study subjects and dosimetry methodologies. Organ-absorbed dose estimations were heterogeneous even among studies using ^68^Ga-labelled bombesin analogues [30, 33, 34]. Absorbed doses of single organs varied greatly between the present study and that of Zhang et al. on the ^68^Ga-labelled GRPR agonist NOTA-Aca-BBN [33]. As an example, based on the results obtained with OLINDA/EXM 1.1 (Additional file 2: Table S2), the absorbed dose to the gallbladder was three times higher, whereas the absorbed dose to the pancreas was 368 times lower in [33] than in the present study. In turn, our organ absorbed dose estimations were, on average, about 70 times higher than those of Zhang et al. [33] (full data not shown). Nevertheless, EDs for reference person were similar: 2.76E-02 vs 3.1E-02 mSv/MBq in [33] and the present study, respectively. This can be attributed to the fact that organs with the highest w_T_, such as the gonads (w_T_ = 0.2), received nearly similar doses in both studies.

**Fig. 4:**
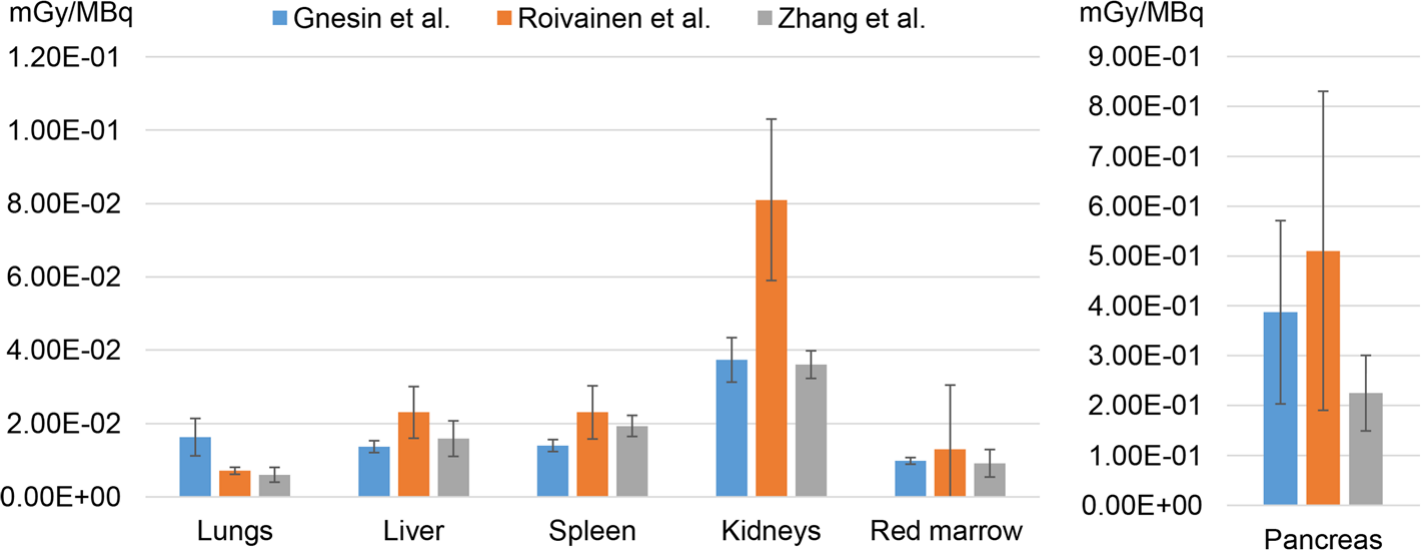
Dosimetry comparison between our study and those of Roivainen et al. [30] and Zhang et al. [34] based on OLINDA/EXM 1.0. The absorbed dose estimations reported in this figure are based on either a 3.5-h urinary voiding cycle (our study and that of Roivainen et al. [30]) or on a 1-h voiding cycle (Zhang et al. [34]). It should be noted that, for the organs reported, variations of urinary voiding cycle do not produce significant changes of absorbed dose estimations. Error bars represent ± 2SD

Although obtained on healthy individuals, the organ-absorbed dose estimations found by Roivainen et al. [30] and by a further publication of Zhang et al. [34] were in the same order of magnitude as ours. These studies were conducted using the ^68^Ga-labelled antagonists DOTA-BAY 86-7548 [30] and NOTA-RM26 [34], respectively. A dosimetry comparison with the present study for most relevant parenchymal organs and bone marrow is given in Fig. 4. In our study, the estimated ED for male was lower than in [30] and [34] (2.79E-02 vs 3.8E-02 vs 6.57E-02 mSv/MBq in the present study, in [30] and in [34], respectively, 1-h voiding cycle).

## Discussion

GRPR, also known as BB2, is receiving great attention as a theranostic target. The most exploited field of application of GRPR-targeting peptides is prostate cancer, although bombesin analogues might be relevant for other highly prevalent tumours [9]. In the work-up of prostate cancer, radiolabelled bombesin analogues might complement other diagnostic probes, such as ^18^F-choline, ^11^C-acetate or ^68^Ga-PSMA, or even compete with them [15, 38]. As regards therapeutic applications, radiolabelled bombesin analogues might be advantageous because of the lack of significant uptake by the lacrimal or salivary glands, which is of serious concern in radionuclide therapy with PSMA [39].

Among several GRPR-targeting imaging probes, the statin-based antagonist ^68^Ga-NODAGA-MJ9 has shown favourable binding properties and has the advantage of being labelled with high efficiency at room temperature [16]. In the present study, we have assessed the dosimetry of ^68^Ga-NODAGA-MJ9 in five patients with relapsing prostate adenocarcinoma. We discuss our results below, with a particular focus on the comparison with similar studies on bombesin analogues and on other theragnostic peptides.

The single organ receiving the highest absorbed dose was the pancreas (260 μGy/MBq). This is in agreement with the notion that pancreas is probably the organ expressing the highest amount of GRPR [5], and in line with previous studies on biodistribution and dosimetry of radiolabelled bombesin analogues in mice [40] and in humans [30, 31, 34].

Although relatively high, such absorbed doses are unlikely to produce clinically relevant pancreatic toxicities in case of therapeutic administrations of radiolabelled bombesin analogues. In fact, we estimated that the absorbed dose to the pancreas would be 1.85 Gy for the administration of a theoretical ^177^Lu-labelled MJ9 compound given at a standard therapeutic activity of 7.4 GBq (Additional file 3: Table S3). This theoretical estimation, although artificially obtained from our measured ^68^Ga-based organ biokinetics, can be considered largely safe as no relevant pancreatic toxicity has been observed for absorbed doses that are at least one order of magnitude higher in external beam radiotherapy which, although not optimal, is presently our only term of comparison [41, 42].

Of note, two previous PET-based dosimetry studies reported substantially inferior radiation absorbed doses to the pancreas after the injection of ^18^F-BAY 864367 [32] and ^68^Ga-NOTA-Aca-BBN [33] (14.36 μGy/MBq and 1.05 μGy/MBq, respectively). These results are questionable, as the pancreas clearly shows a prominent uptake on the PET images featured by these publications [32, 33]. In the striking case of [33], the radiation-absorbed dose reported for the pancreas was even lower than that reported for the muscle (1.39 μGy/MBq). These important discrepancies are unlikely justified by different tracer kinetics; rather they are probably due to different methodologies used for dose calculations. This highlights the need for a standardisation of methodology and data reporting in clinical dosimetry procedures.

The absorbed dose to the kidneys resulted to be 34.8 μGy/MBq for ^68^Ga-NODAGA-MJ9, which is lower than that estimated for other ^68^Ga-labelled peptides, such as PSMA or somatostatin analogues [43–47]. Overall, these studies showed absorbed doses to the kidneys ranging from 89 to 262 μGy/MBq [43–47].

In the present study, by assuming a urinary voiding interval of 1 h, we calculated an ED of 18.8 μSv/MBq in men, which would correspond to 2.8 mSv after a tracer injection of 150 MBq ^68^Ga-NODAGA-MJ9. This estimated ED falls within the range 16.7–25.7 μSv/MBq reported for other ^68^Ga-labelled peptides [43–47]. However, differently from previous studies, our absorbed dose calculations were based on OLINDA/EXM 2.0. If the absorbed dose estimation is made with OLINDA/EXM 1.1, the ED in male increases up to 27.9 μSv/MBq for ^68^Ga-NODAGA-MJ9. The lower ED estimated with OLINDA/EXM 2.0 can be explained, in first instance, by a lower tissue w_T_ attributed to the most irradiated organ, that is the pancreas in our case (w_T_ pancreas = 0.025 vs. 0.0092 in OLINDA/EXM 1.1 and OLINDA/EXM 2.0, respectively) [24]. Secondarily, the phantoms implemented in OLINDA/EXM 1.1 and OLINDA/EXM 2.0 consider two different organ masses for the pancreas (94.3 g vs. 140 g in OLINDA/EXM 1.1 and OLINDA/EXM 2.0 respectively), which has an additional impact on the calculation of the ED. Consequently, in our specific case of ^68^Ga-NODAGA-MJ9, the equivalent dose to the pancreas accounts for 35% of the ED in OLINDA/EXM 1.1, whereas it accounts for only 13% of the ED in OLINDA/EXM 2.0.

As discussed by some authors [43, 48], in OLINDA/EXM 2.0, EDs would be lower compared to OLINDA/EXM 1.1 for both ^68^Ga-labelled PSMAs and somatostatin analogues, as well. Therefore, whatever version of OLINDA/EXM is used, ED is slightly higher for ^68^Ga-NODAGA-MJ9 than for ^68^Ga-labelled PSMAs or somatostatin analogues.

This study has inherent limitations due to the small number of patients. Moreover, by chance, one patient had history of pancreatic carcinoma treated with pancreaticoduodenectomy, and two additional patients had their gallbladder surgically removed for gallstones. This has further reduced the number of our observations.

In addition, dosimetry data regarding prostate gland have probably little clinical significance in the context of our study. In fact, we enrolled only patients who had their prostate surgically removed, so that absorbed doses to the prostate have been estimated by the software considering only the contribution of neighbouring organs as sources of radiation. Analogously, our extrapolation to female dosimetry should be interpreted with caution, especially concerning the estimated absorbed dose to breast and reproductive organs. Nevertheless, the dosimetry calculation for female subject is required in order to calculate the ED which, according to the ICRP103 methodology [24], is based on both male and female organ equivalent dose ponderation.

## Conclusions

We have performed the first human dosimetry of ^68^Ga-NODAGA-MJ9. Our data shows the pancreas to be the most irradiated organ in ^68^Ga-NODAGA-MJ9 diagnostic procedures, followed by the urinary bladder wall, the small intestine, and the kidneys. ED is in the same range of other common ^68^Ga-labelled peptides. The differences of dosimetry results between the present study and other previous works on radiolabelled bombesin analogues mainly depend on the methodology used for absorbed dose calculations.

## Additional files

Additional file 1: **Table S1.** Patient-specific dosimetry for the five male subjects included in the study (1-h urinary voiding cycle). (DOCX 25 kb)

Additional file 2: **Table S2.** Extrapolated organ absorbed doses and ED according to OLINDA/EXM 1.1 for 1-h and 3.5 h urinary voiding cycles in male, female and the reference person. (DOCX 19 kb)

Additional file 3: **Table 3.** Extrapolated absorbed doses to the pancreas per administered GBq of a theoretical ^177^Lu-MJ9 analogue. (DOCX 20 kb)
