# Supplementary material for: First in-human radiation dosimetry of the gastrin-releasing peptide (GRP) receptor antagonist 68Ga-NODAGA-MJ9
Source: EJNMMI Res. 2018 Dec 12;8:108. doi: 10.1186/s13550-018-0462-9 (PMC6291411; doi:10.1186/s13550-018-0462-9)
Supplement: Supplementary file 1 — Table S1. Patient-specific dosimetry for the five male subjects included in the study (1-h urinary voiding cycle). (DOCX 25 kb) [file 13550_2018_462_MOESM1_ESM.docx]

**Table S1**  Patient-specific dosimetry for the five male subjects included in the study (1-h urinary voiding cycle).

Source organ masses (source organs: brain, thyroid, lungs, liver, adrenals, spleen, pancreas, kidneys, heart, stomach, intestines, gallbladder and urinary bladder) were obtained by manual segmentation of CT data. The masses of remaining target organs were obtained by multiplying the reference phantom organ masses by the ratio: patient’s weight / 73 Kg. This mass rescaling tool is available in OLINDA/EXM 2.0. Source organ TIACs were obtained as described in the material and methods section of the article. Patient-specific organ absorbed doses were, on average, 8.8% lower than those obtained using the reference organ masses.

| Patient n° | P1 | P2 | P3 | P4 | P5 | mean | SD |
| --- | --- | --- | --- | --- | --- | --- | --- |
| Height (m) | 1.72 | 1.83 | 1.81 | 1.88 | 1.78 | 1.804 | 0.06 |
| Body mass (kg) | 93 | 74 | 90 | 88 | 77 | 84 | 83 |
| Target organ mass (g) | | | | | | | |
| Adrenals | 13.5 | 13.5 | 18.7 | 14.8 | 11.3 | 14.4 | 2.7 |
| Brain | 1454.3 | 1766.1 | 1597.1 | 1387.1 | 1602.3 | 1561.4 | 147.2 |
| Gallbladder wall | 12.7 | - | - | - | 10.5 | 11.6 | 1.5 |
| Left colon | 191.1 | 152.1 | 184.9 | 180.8 | 158.2 | 173.4 | 17.2 |
| Small Intestine | 828.1 | 658.9 | 801.4 | 783.6 | 685.6 | 751.5 | 74.7 |
| Stomach wall | 191.1 | 152.1 | 184.9 | 180.8 | 158.2 | 173.4 | 17.2 |
| Right Colon | 191.1 | 152.1 | 184.9 | 180.8 | 158.2 | 173.4 | 17.2 |
| Rectum | 89.2 | 71.0 | 86.3 | 84.4 | 73.8 | 80.9 | 8.0 |
| Heart Wall* | 420.4 | 334.5 | 406.8 | 397.8 | 348.1 | 381.5 | 37.9 |
| Kidneys | 493.5 | 423.2 | 526.1 | 494.6 | 392.7 | 466.0 | 55.6 |
| Liver | 2254.4 | 1537.2 | 1940.4 | 1765.1 | 1778.7 | 1855.1 | 265.4 |
| Lungs | 1528.8 | 1216.4 | 1479.5 | 1446.6 | 1265.8 | 1387.4 | 137.8 |
| Pancreas | 163.8 | - | 132.3 | 140.7 | 162.8 | 149.9 | 15.8 |
| Red Marrow | 1490.5 | 1186.0 | 1442.5 | 1410.4 | 1234.1 | 1352.7 | 134.4 |
| Spleen | 275.6 | 194.5 | 202.8 | 211.1 | 195.5 | 215.9 | 34.0 |
| Thyroid | 9.4 | 10.1 | 18.3 | 18.4 | 15.4 | 14.3 | 4.4 |
| Urinary bladder wall | 63.7 | 50.7 | 61.6 | 60.3 | 52.7 | 57.8 | 5.7 |
| organ TIAC (MBq.h/MBq) | | | | | | | |
| Adrenals | 1.34E-04 | 3.28E-04 | 1.96E-04 | 2.52E-04 | 3.31E-04 | 2.48E-04 | 8.49E-05 |
| Brain | 1.37E-03 | 3.06E-03 | 6.21E-04 | 2.05E-03 | 5.67E-04 | 1.53E-03 | 1.05E-03 |
| Gallbladde cont | 2.70E-03 | - | - | - | 4.50E-03 | 3.60E-03 | 1.27E-03 |
| Left colon | 3.59E-03 | 1.15E-02 | 8.81E-03 | 5.56E-03 | 4.21E-03 | 6.73E-03 | 3.33E-03 |
| Small Intestine | 4.17E-02 | 6.82E-02 | 2.56E-02 | 3.82E-02 | 2.70E-02 | 4.01E-02 | 1.71E-02 |
| Stomach content | 4.71E-03 | 8.84E-03 | 4.38E-03 | 3.72E-03 | 2.30E-03 | 4.79E-03 | 2.45E-03 |
| Right Colon | 7.17E-03 | 2.29E-02 | 1.76E-02 | 1.11E-02 | 8.42E-03 | 1.35E-02 | 6.66E-03 |
| Rectum | 3.59E-03 | 1.15E-02 | 8.81E-03 | 5.56E-03 | 4.21E-03 | 6.73E-03 | 3.33E-03 |
| Heart Content | 1.72E-02 | 2.35E-02 | 1.71E-02 | 3.02E-02 | 2.01E-02 | 2.16E-02 | 5.45E-03 |
| Kidneys | 1.93E-02 | 2.50E-02 | 2.12E-02 | 2.17E-02 | 1.99E-02 | 2.14E-02 | 2.23E-03 |
| Liver | 3.66E-02 | 3.78E-02 | 3.41E-02 | 3.93E-02 | 4.20E-02 | 3.79E-02 | 2.93E-03 |
| Lungs | 2.38E-02 | 3.48E-02 | 2.94E-02 | 3.42E-02 | 2.16E-02 | 2.88E-02 | 5.98E-03 |
| Pancreas (CT) | 9.40E-02 | - | 8.26E-02 | 5.33E-02 | 9.53E-02 | 8.13E-02 | 1.95E-02 |
| Red Marrow | 6.83E-03 | 8.54E-03 | 7.03E-03 | 6.24E-03 | 6.86E-03 | 7.10E-03 | 8.59E-04 |
| Spleen | 2.94E-03 | 3.93E-03 | 2.47E-03 | 3.81E-03 | 2.64E-03 | 3.16E-03 | 6.73E-04 |
| Thyroid | 3.31E-04 | 3.49E-04 | 2.32E-04 | 3.56E-04 | 3.29E-04 | 3.20E-04 | 5.00E-05 |
| Urinary bladder cont | 1.07E-01 | 6.20E-02 | 8.67E-02 | 8.52E-02 | 9.79E-02 | 8.77E-02 | 1.69E-02 |
| Rest of body | 1.04E+00 | 1.00E+00 | 1.13E+00 | 9.80E-01 | 1.08E+00 | 1.05E+00 | 5.90E-02 |
| Absorbed doses (mGy/MBq) | | | | | | | |
| Adrenals | 9.32E-03 | 1.52E-02 | 9.27E-03 | 1.19E-02 | 1.68E-02 | 1.25E-02 | 3.42E-03 |
| Brain | 1.64E-03 | 1.92E-03 | 1.39E-03 | 1.87E-03 | 1.32E-03 | 1.63E-03 | 2.72E-04 |
| Esophagus | 8.69E-03 | 9.88E-03 | 9.50E-03 | 8.74E-03 | 1.05E-02 | 9.46E-03 | 7.70E-04 |
| Eyes | 6.70E-03 | 7.95E-03 | 7.48E-03 | 6.64E-03 | 8.24E-03 | 7.40E-03 | 7.21E-04 |
| Gallbladder Wall | 2.06E-02 | 1.19E-02 | 1.11E-02 | 1.00E-02 | 3.03E-02 | 2.55E-02 | 6.86E-03 |
| Left colon | 2.28E-02 | 4.56E-02 | 3.86E-02 | 2.73E-02 | 2.68E-02 | 3.22E-02 | 9.51E-03 |
| Small Intestine | 3.78E-02 | 5.59E-02 | 2.82E-02 | 3.49E-02 | 3.05E-02 | 3.75E-02 | 1.10E-02 |
| Stomach Wall | 1.79E-02 | 1.87E-02 | 1.80E-02 | 1.50E-02 | 1.82E-02 | 1.76E-02 | 1.46E-03 |
| Right colon | 2.05E-02 | 4.60E-02 | 3.71E-02 | 2.61E-02 | 2.45E-02 | 3.08E-02 | 1.05E-02 |
| Rectum | 2.11E-02 | 4.61E-02 | 3.72E-02 | 2.65E-02 | 2.48E-02 | 3.11E-02 | 1.03E-02 |
| Heart Wall | 1.70E-02 | 2.13E-02 | 1.78E-02 | 2.33E-02 | 2.04E-02 | 2.00E-02 | 2.58E-03 |
| Kidneys | 2.34E-02 | 3.23E-02 | 2.44E-02 | 2.56E-02 | 2.81E-02 | 2.68E-02 | 3.56E-03 |
| Liver | 1.19E-02 | 1.52E-02 | 1.26E-02 | 1.45E-02 | 1.56E-02 | 1.40E-02 | 1.63E-03 |
| Lungs | 9.93E-03 | 1.51E-02 | 1.20E-02 | 1.34E-02 | 1.07E-02 | 1.22E-02 | 2.08E-03 |
| Pancreas | 2.70E-01 | 1.22E-02 | 2.74E-01 | 1.71E-01 | 2.75E-01 | 2.00E-01 | 1.14E-01 |
| Prostate | 1.13E-02 | 1.23E-02 | 1.19E-02 | 1.07E-02 | 1.32E-02 | 1.19E-02 | 9.55E-04 |
| Salivary Glands | 7.32E-03 | 8.65E-03 | 8.17E-03 | 7.26E-03 | 8.97E-03 | 8.07E-03 | 7.71E-04 |
| Red Marrow | 6.98E-03 | 9.20E-03 | 7.70E-03 | 7.02E-03 | 9.06E-03 | 7.99E-03 | 1.08E-03 |
| Osteogenic Cells | 5.38E-03 | 6.69E-03 | 5.91E-03 | 5.30E-03 | 6.69E-03 | 5.99E-03 | 6.77E-04 |
| Spleen | 8.87E-03 | 1.31E-02 | 9.89E-03 | 1.22E-02 | 1.05E-02 | 1.09E-02 | 1.72E-03 |
| Testes | 8.04E-03 | 9.15E-03 | 8.80E-03 | 7.83E-03 | 9.73E-03 | 8.71E-03 | 7.85E-04 |
| Thymus | 8.11E-03 | 9.73E-03 | 8.99E-03 | 8.47E-03 | 9.94E-03 | 9.05E-03 | 7.87E-04 |
| Thyroid | 1.49E-02 | 1.46E-02 | 7.84E-03 | 9.89E-03 | 1.08E-02 | 1.16E-02 | 3.07E-03 |
| Urinary Bladder Wall | 1.29E-01 | 8.13E-02 | 1.08E-01 | 1.05E-01 | 1.23E-01 | 1.09E-01 | 1.86E-02 |
| Total Body | 9.06E-03 | 1.04E-02 | 9.84E-03 | 8.88E-03 | 1.09E-02 | 9.82E-03 | 8.61E-04 |
| ED (ICRP 103) mSv/MBq | 1.75E-02 | 1.77E-02 | 1.87E-02 | 1.62E-02 | 1.85E-02 | 1.77E-02 | 9.78E-04 |
