# Supplementary material for: First in-human radiation dosimetry of the gastrin-releasing peptide (GRP) receptor antagonist 68Ga-NODAGA-MJ9
Source: EJNMMI Res. 2018 Dec 12;8:108. doi: 10.1186/s13550-018-0462-9 (PMC6291411; doi:10.1186/s13550-018-0462-9)
Supplement: Supplementary file 2 — Table S2. Extrapolated organ absorbed doses and ED according to OLINDA/EXM 1.1 for 1-h and 3.5 h urinary voiding cycles in male, female and the reference person. (DOCX 19 kb) [file 13550_2018_462_MOESM2_ESM.docx]

**Table S2** Extrapolated organ absorbed doses and ED according to OLINDA/EXM 1.1 for 1-h and 3.5h urinary voiding cycles in male, female and the reference person.

|  | 1h-voiding | | | 3.5h-voiding | | |
| --- | --- | --- | --- | --- | --- | --- |
|  | Organ dose (mGy/MBq) | | | Organ dose (mGy/MBq) | | |
| Organ | Male | Female | Reference Person | Male | Female | Reference Person |
| Adrenals | 1.25E-02 | 1.50E-02 | 1.38E-02 | 1.27E-02 | 1.51E-02 | 1.39E-02 |
| Brain | 1.66E-03 | 2.08E-03 | 1.87E-03 | 1.69E-03 | 2.12E-03 | 1.91E-03 |
| Breasts | 8.50E-03 | 1.09E-02 | 9.70E-03 | 8.74E-03 | 1.12E-02 | 9.97E-03 |
| Gallbladder Wall | 2.63E-02 | 3.07E-02 | 2.85E-02 | 2.67E-02 | 3.11E-02 | 2.89E-02 |
| LLI Wall | 2.94E-02 | 3.37E-02 | 3.16E-02 | 3.08E-02 | 3.54E-02 | 3.31E-02 |
| Small Intestine | 3.23E-02 | 3.77E-02 | 3.50E-02 | 3.30E-02 | 3.87E-02 | 3.59E-02 |
| Stomach Wall | 1.65E-02 | 2.01E-02 | 1.83E-02 | 1.68E-02 | 2.05E-02 | 1.87E-02 |
| ULI Wall | 2.65E-02 | 3.13E-02 | 2.89E-02 | 2.71E-02 | 3.20E-02 | 2.96E-02 |
| Heart Wall | 2.16E-02 | 2.57E-02 | 2.37E-02 | 2.19E-02 | 2.61E-02 | 2.40E-02 |
| Kidneys | 3.72E-02 | 4.08E-02 | 3.90E-02 | 3.73E-02 | 4.10E-02 | 3.92E-02 |
| Liver | 1.36E-02 | 1.80E-02 | 1.58E-02 | 1.37E-02 | 1.81E-02 | 1.59E-02 |
| Lungs | 1.63E-02 | 2.03E-02 | 1.83E-02 | 1.63E-02 | 2.04E-02 | 1.84E-02 |
| Muscle | 9.66E-03 | 1.22E-02 | 1.09E-02 | 1.02E-02 | 1.29E-02 | 1.16E-02 |
| Ovaries | — | 1.56E-02 | 1.56E-02 | — | 1.73E-02 | 1.73E-02 |
| Pancreas | 3.87E-01 | 4.29E-01 | 4.08E-01 | 3.87E-01 | 4.29E-01 | 4.08E-01 |
| Red Marrow | 9.42E-03 | 1.10E-02 | 1.02E-02 | 9.81E-03 | 1.15E-02 | 1.07E-02 |
| Osteogenic Cells | 1.39E-02 | 1.88E-02 | 1.64E-02 | 1.43E-02 | 1.95E-02 | 1.69E-02 |
| Skin | 8.14E-03 | 1.03E-02 | 9.22E-03 | 8.47E-03 | 1.07E-02 | 9.59E-03 |
| Spleen | 1.39E-02 | 1.68E-02 | 1.54E-02 | 1.40E-02 | 1.70E-02 | 1.55E-02 |
| Testes | 9.71E-03 | — | 9.71E-03 | 1.07E-02 | — | 1.07E-02 |
| Thymus | 9.67E-03 | 1.24E-02 | 1.10E-02 | 9.93E-03 | 1.27E-02 | 1.13E-02 |
| Thyroid | 9.51E-03 | 1.12E-02 | 1.04E-02 | 9.59E-03 | 1.13E-02 | 1.04E-02 |
| Urinary Bladder Wall | 1.10E-01 | 1.47E-01 | 1.29E-01 | 2.08E-01 | 2.79E-01 | 2.44E-01 |
| Uterus | — | 1.65E-02 | 1.65E-02 | — | 1.97E-02 | 1.97E-02 |
| Total Body | 1.11E-02 | 1.41E-02 | 1.26E-02 | 1.16E-02 | 1.48E-02 | 1.32E-02 |
| ED (ICRP-60) mSv/MBq | 2.79E-02 | 3.41E-02 | 3.10E-02 | 3.11E-02 | 4.15E-02 | 3.63E-02 |
