# Supplementary material for: First in-human radiation dosimetry of the gastrin-releasing peptide (GRP) receptor antagonist 68Ga-NODAGA-MJ9
Source: EJNMMI Res. 2018 Dec 12;8:108. doi: 10.1186/s13550-018-0462-9 (PMC6291411; doi:10.1186/s13550-018-0462-9)
Supplement: Supplementary file 3 — Table 3. Extrapolated absorbed doses to the pancreas per administered GBq of a theoretical 177Lu-MJ9 analogue. (DOCX 20 kb) [file 13550_2018_462_MOESM3_ESM.docx]

**Table 3** Extrapolated absorbed doses to the pancreas per administered GBq of a theoretical ^177^Lu-MJ9 analogue.

| **Organ** | T biol. (h) | T eff. (h) | TIAC (h) | ^177^Lu- MJ9 (extr.) (Gy/GBq) |
| --- | --- | --- | --- | --- |
| Pancreas | 4.81 | 4.67 | 4.06E-01 | 2.50E-01 |

Effective half-life (T_e_Ga-68_) of ^68^Ga-MJ9 was obtained from mono-exponential fit on measured organ time activity data. Biological half-life (T_b_) was computed by the formula: T_b_ = T_e_Ga68_ × T_p_Ga68_ / (T_p_Ga-68_ - T_e_Ga-68_), where T_p_Ga-68_ = 1.13 h is the physical half-life of ^68^Ga. The effective half-life of ^177^Lu-MJ9 was obtained by assuming a physical half-life of ^177^Lu equal to 160.8 h. The TIAC was obtained by analytical integration as follows: $AC= \int_{t_{admin}}^{\infty} \frac{A\left( t \right)}{\left( A_{admin} \right)}dt= \frac{A\left( 0 \right)}{\left( A_{admin} \right)}\times\left( \frac{\ln\left( 2 \right)}{T_{e_{Lu-177}}} \right)^{-1}$.

The TIAC was used as kinetic input in OLINDA/EXM 2.0 to obtain the delivered dose in Gy/GBq. As a result, the absorbed dose to the pancreas would be 1.85 Gy for the administration of a theoretical ^177^Lu-labeled MJ9 compound given at a standard therapeutic activity of 7.4GBq.
